# Microscale Simulation on Mechanical Properties of Al/PTFE Composite Based on Real Microstructures

**DOI:** 10.3390/ma9070590

**Published:** 2016-07-19

**Authors:** Chao Ge, Yongxiang Dong, Wubuliaisan Maimaitituersun

**Affiliations:** State Key Laboratory of Explosion Science and Technology, Beijing Institute of Technology, Beijing 100081, China; gechao@bit.edu.cn (C.G.); 2120140256@bit.edu.cn (W.M.)

**Keywords:** polymer composites, Al/PTFE, finite-element simulation, microscale modelling, quasi-static compression

## Abstract

A novel numerical method at the microscale for studying the mechanical behavior of an aluminum-particle-reinforced polytetrafluoroethylene (Al/PTFE) composite is proposed and validated experimentally in this paper. Two types of 2D representative volume elements (RVEs), real microstructure-based and simulated microstructures, are established by following a series of image processing procedures and on a statistical basis considering the geometry and the distribution of particles and microvoids, respectively. Moreover, 3D finite element modelling based on the same statistical information as the 2D simulated microstructure models is conducted to show the efficiency and effectiveness of the 2D models. The results of all simulations and experiments indicate that real microstructure-based models and simulated microstructure models are efficient methods to predict elastic and plastic constants of particle-reinforced composites.

## 1. Introduction

Impact-initiated composite material, characterized by its exothermic and rapid energy releasing property upon impact with targets or when impacted, has been of concern in recent years. It can be categorized in a much larger category—i.e., reactive materials (RMs), which denotes the class of materials generally combining two or more non-explosive solids that, upon their ignition, react to release chemical energy in addition to the kinetic energy resulting when the high-speed projectiles containing the reactive materials collide with the target [1]. Impact-initiated composites are typically formed by means of pressing/sintering uniformly mixed active metal powders into a polymer matrix, which will introduce sufficient strength and insensitivity; thus, they have a number of potential ordnance applications.

Among the many formulas of impact-initiated composites, aluminum (Al)-particle-filled polytetrafluoroethylene (PTFE) is typical, which has become a benchmark for investigation into properties of impact-initiated composites. Based on the basic formulas of Al/PTFE, over the past decades, much progress has been achieved, especially in formulations and fabrications, mechanical properties of both statics and dynamics, flow and failure, impact initiation mechanisms and properties, and reaction and energy release properties [2,3,4,5,6,7,8,9,10,11]. All conducted research improves the explanation of Al/PTFE while promoting its application and improvement.

However, all research has been conducted macroscopically; microstructural properties and the effects of a microstructure on its macro properties and performance are still less known. From the perspective of fabrication and application of Al/PTFE, different particle geometries, volume fractions and distributions may result in different composite properties, and the pressing/sintering process may introduce microvoids and microcracks, which will affect the initiation properties and strength of the composite. Moreover, the demand in the application of Al/PTFE is proposed with a higher density, better strength, sufficient insensitivity and complete reaction. All of these properties depend on the microstructural characteristics of Al/PTFE and cannot be neglected.

With the development of algorithms and modelling techniques, finite element analysis (FEA) is increasingly adopted in mechanics of materials both macroscopically and microscopically. Especially with the combination of trans-scale mechanics and FEA technology, the mechanical properties of materials are being understood at increasingly smaller scales [12,13]. In this context, micromechanical analyses have been on an increasing trend to understand the behavior of modern materials with sophisticated microstructures—e.g., fiber- or particle-reinforced composites and textile composites. Resorting to the concept of unit cells and representative volume elements (RVE), many studies have been conducted on particle-reinforced polymer/metal matrix composites [14,15]. Li [16] established unit cells and boundary conditions for a range of typical packing systems (simple cubic packing, body-centered cubic packing, face-centered cubic packing, and close-packed hexagonal packing) and examined them in a systematic manner with translational symmetry transformations, which was then widely referenced in investigation into many types of particle-reinforced composites. Huang and Bush [17] modeled the indentation process of aluminum–aluminum composites by a cylindrical unit cell and a hexagonal arrangement to investigate the elastoplastic behavior, and the results demonstrated the influence of grain size on the mechanical properties of the composites. Based on the concept of RVE, Sun [18] determined the appropriate boundary conditions on the RVE for various loading conditions by using symmetry and periodicity conditions; elastic constants predicted by finite element analysis showed good agreement with existing theoretical predictions and available experimental data. Song [19] established a multi-particle unit cell model to investigate the mechanical characteristics and the fracture behavior of 91W-6.3Ni-2.7Fe tungsten alloys, with the results of modelling showing good agreement with that of an in situ tensile test.

However, both unit cell and RVE models are highly idealized models for heterogeneous materials because all materials are regarded as regular particle arrangements or even particles of the same geometries. The unit cell approach assumes that the composite is constructed of an array of basic units, each with identical composition, geometry, inclusion shape and material properties [20]. As a matter of fact, microstructures of multiphase materials mostly have strong stochastic characteristics. Microstructural parameters, such as geometry, distribution, volume fraction, and sizes and orientations of reinforcement particles, are all random to a certain degree and affect the mechanical properties of composites. In some cases, microvoids and microcracks introduced during fabrication may become key factors in material failure, fracture and even initiation and reaction of reactive materials. To overcome the drawbacks of unit cell models and RVE (especially highly idealized) models, simulated microstructures of particle-reinforced composites based on certain statistical information from the original structure are developed, with which randomness of particle distribution, particle gradation and shapes can be accounted. Simulated microstructures are RVEs as well, but they are more practical RVEs. Wang [21] determined the elastic properties of multiphase composites by proposing a random-generation growth (RGG) method coupled with a lattice Boltzmann method to reproduce the random microstructure. Guo [22] studied the mechanical response of incompressible particle-reinforced neo-Hookean (IPRNC) composites by creating three-dimensional simulated RVE models assuming the randomly distributed particles to be spheres, and mechanical response of IPRNC was well predicted. Leite [23] proposed a numerical algorithm that allows the control of the aggregate volume fraction, shape and degradation to generate 2D and 3D models of the heterogeneous internal structure of cementitious composites and simulated the fracture process. Some other researchers studied particle-reinforced composites by a random generation method assuming particles as spheres, ellipsoids or regular polyhedrons, considering particle distribution, degradation and shape effects, and this method was proved to be robust under certain circumstances, though not sufficiently [24,25,26,27,28,29].

Given the significant influences of the geometric details on the material properties, methods aimed at approaching the real structures must be proposed. Given that the microscopy instruments and observation technologies are now well developed, image mapping has become a highly powerful tool for this purpose [30]. Two-dimensional finite element models of real structures are usually built based on X-ray CT (Computed Tomography), optical microscopy and scanning electron microscopy (SEM) images processed by digital image processing techniques. Three-dimensional element models of real structures are usually reconstructed by a serial sectioning process. By this method, inherent particle morphology and microstructural characteristics of composites are considered, and accurate simulation results are yielded. To this point, real microstructure-based RVEs can be regarded as the most practical RVEs.

In this paper, we present a comprehensive study of two-dimensional real microstructure-based finite element modelling of the deformation behavior of aluminum (Al)-particle-filled polytetrafluoroethylene (PTFE); such studies are considerably limited at a microscopic level. In addition to this, FE models from statistics-based simulated microstructures considering microvoids are established to draw comparisons. All simulated results are compared with experimental results.

## 2. Static Material Experiments

The samples studied in this paper are a pressed and sintered mixture of Al and PTFE powders, 26.5% and 73.5% by weight, respectively. The samples were fabricated by the authors themselves, based on Joshi’s [2] patent, and is outlined below.

**Figure 1:**
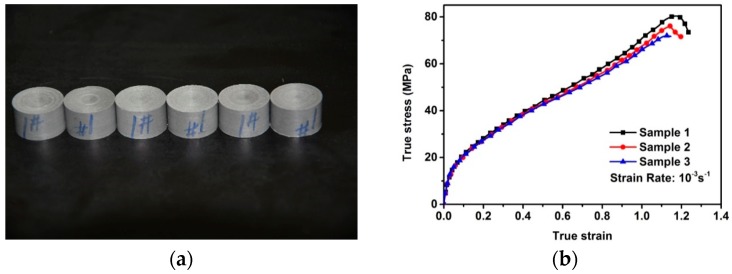
(**a**) Prepared Al/PTFE (polytetrafluoroethylene) samples and (**b**) true stress–strain curves of samples under quasi-static compression.

First, powder of Al (Hunan Goldsky Aluminum Industry High-Tech Co., Ltd., Changsha, China, JT-4) and PTFE (DuPont PTFE 9002-84-0, type MP 1000) was mixed in proportions of 26.5% to 73.5% by weight, respectively, via a dry mixing process. After the mixture was made, the material was pressed in a die to make a flat cylindrical sample 60 mm in diameter and 15 mm in height. Pressure applied to the mixture in the die was in the range of 70 MPa to 80 MPa with a dwell time of approximately 10 min. The pressed mixture then underwent a sintering cycle to prevent any oxidation or surface reaction of the material, which was performed under an argon atmosphere. The sample was heated at a rate of approximately 50 degrees per hour to a final temperature of approximately 380 °C, held at this final temperature for 4 h, and then cooled to room temperature via an initially slow and then fast process. Figure 1a shows a picture of prepared Al/PTFE samples with dimensions of Φ 12.00 mm × 10.00 mm, turned from the original flat cylindrical sample.

To validate the FE models and algorithm, quasi-static compression tests with the employment of an Instron, Inc. (Norwood, MA, USA), model 5985 servo-hydraulic load frame, were performed in a standard laboratory environment (23 ± 2 °C, 30%–40% relative humidity). The load applied to specimens was measured with a load cell mounted to the crosshead. The tests were carried out under displacement control with a constant crosshead speed of 0.6 mm/min, which corresponds to a nominal strain rate of 10^−3^·s^−1^. Contact surfaces of specimens were lubricated before the test. For reliable results, three samples were tested. The true stress–strain curves of three tested specimens under quasi-static compression are shown in Figure 1b. The stress–strain curves of the Al/PTFE samples showed good repeatability. Linear elastic, linear-strain hardening and strain softening stages can be observed, indicating that it is a typical hard and tough composite. From the curves, elastic–plastic constants, including elastic modulus, yield strength, yield strain and plastic hardening modulus can be calculated to characterize the elastic–plastic behavior of this material.

## 3. Real Microstructures and Simulated Microstructures

Microscopic images of Al/PTFE were obtained by a HITACHI S-4800 scanning electron microscope (Tokyo, Japan). Five images of different magnifications were chosen (Table 1). The dimensions of the five microstructures are 316 μm × 221 μm (1); 316 μm × 221 μm (2); 140 μm × 98 μm (3); 97 μm × 68 μm (4); and 84 μm × 59 μm (5). From the images, aluminum particles (white), matrix (grey) and microvoids (black) can be observed. The microvoids were introduced during the pressing/sintering process. Microvoids of diameters in a certain range were distributed randomly, which would have a great effect on the composite properties and cannot be neglected.

### 3.1. Real Microstructure-Based Models

The SEM images then must be processed via an image processing method, of which the main aim is to extract the edges of the particles and microvoids [31,32]. The process was realized by a MATLAB program (V7.13, Mathworks, Natick, MA, USA) developed by the author. First, piecewise linear transformation was applied to the SEM images to enhance the contrast among particles, microvoids and matrix. For clear edges, noise reduction and median filtering for the images were then conducted, after which some local noise was eliminated, and the edges between particles, microvoids and matrix were smoother and clearer. Next, the canny operator was employed, and clear edges connected by single pixels were obtained, as shown in the middle column of Table 1. Al particles and microvoids are represented by white hollow geometries and black solid geometries, respectively. Finally, the images of edges were vectorized, converted into DXF (Drawing Interchange Format) format and imported into the finite element analysis software program ABAQUS (V6.11, Providence, RI, USA). The five real microstructure-based models were marked as 1-R, 2-R, 3-R, 4-R and 5-R, respectively, with R indicating “real.”

### 3.2. Simulated Microstructure Characterization and Reproduction Algorithm

**Figure 2:**
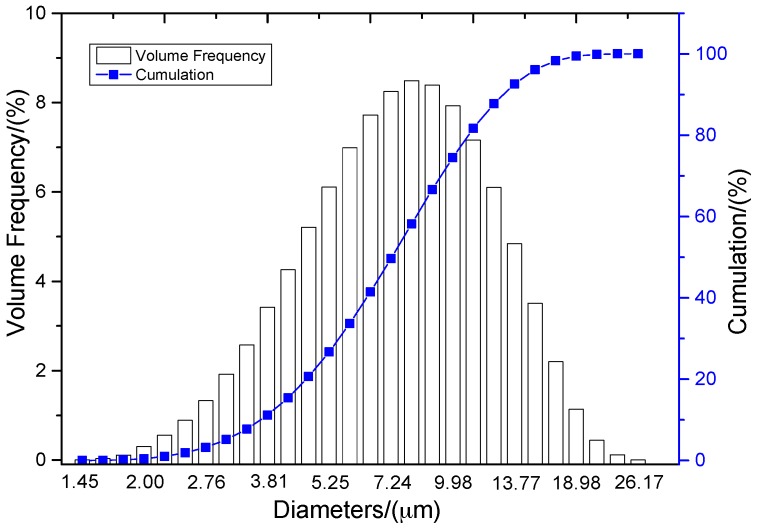
Distribution of aluminum particle diameters.

Simulated microstructures of Al/PTFE were set up based on the statistics of particles and voids and fulfilled by another MATLAB program. The particle size distribution of Al is depicted in Figure 2, which was supplied by the manufacturer, Hunan Goldsky Aluminum Industry High-Tech Co., Ltd., Changsha, China.

**Figure 3:**
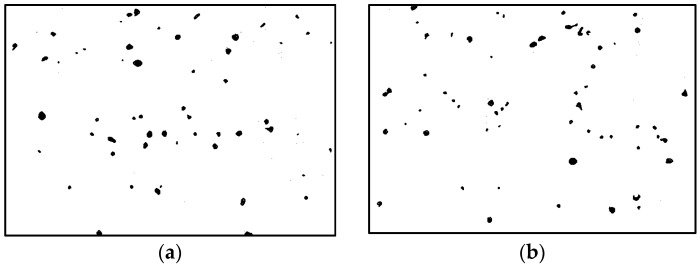
Distribution and geometry of microvoids extracted from SEM images. (**a**) From 1-R; and (**b**) from 2-R microstructure in Table 1.

Statistics on the microvoids were collected based on their distribution and geometry extracted from the SEM images (one and two real structure images in Table 1), as shown in Figure 3.

**Figure 4:**
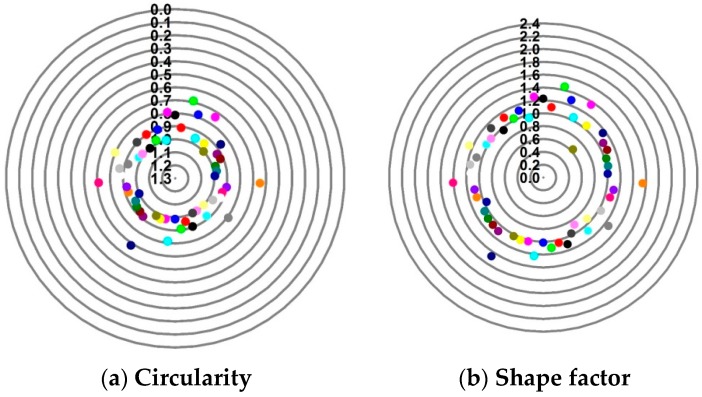
(**a**) Circularity *C*; and (**b**) shape factor *S* of the microvoids extracted from the microstructures.

**Figure 5:**
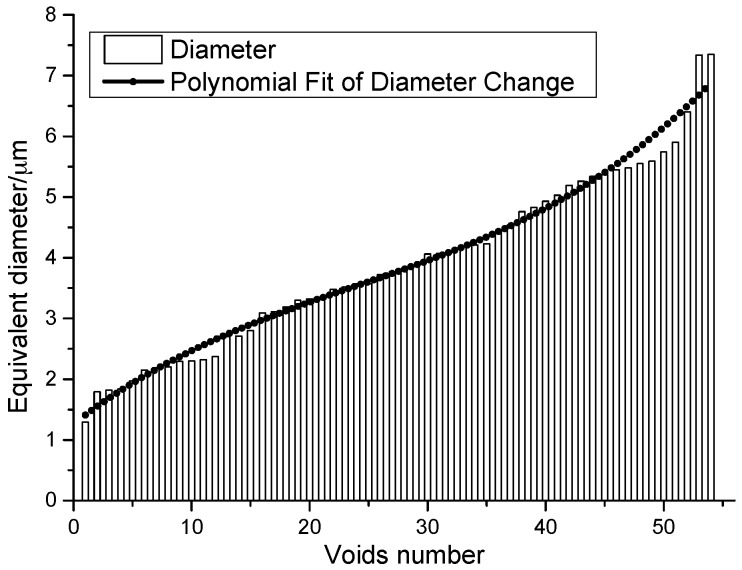
Equivalent diameter distribution of microvoids.

The shapes of microvoids are described by shape factor *S* and circularity *C*:

(1) $$S=\frac{{\text{П}}^{2}}{4\pi A}$$

(2) $$C=4\pi \frac{A}{{\text{П}}^{2}}$$

where П is the perimeter of voids, and *A* is the area of voids. Values of *S* and *C* of 1.0 indicate a perfect circle. As either value approaches 0.0, it indicates an increasingly elongated shape. Statistics of *S* and *C* for the voids are depicted in Figure 4, from which we can see that the circularity of voids is between 0.8 and 1, whereas the shape factor is between 0.8 and 1.2. To this extent, it is reasonable to approximate microvoids as circles, and the equivalent diameter *d_v_* can be calculated following Equation (3). The distribution of equivalent diameters is shown in Figure 5. In addition, the porosity of 1.185% is calculated from the statistical characteristics of voids.

(3) $$d_{v}=\frac{4A}{\text{П}}$$

**Figure 6:**
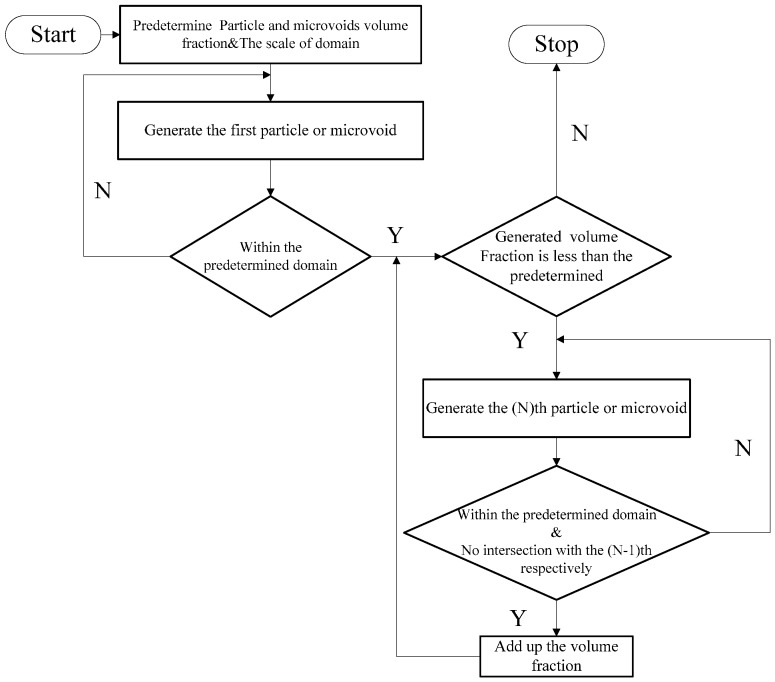
Flowchart of the reproduction process of the simulated microstructure of Al/PTFE.

The reproduction of a simulated microstructure is based on a random sequential adsorption (RSA) algorithm, which was initially used for studying protein adsorption and is widely used for regeneration of composite microstructures [33]. The basic flow of the RSA algorithm consists of generating the first particle and microvoid within the predetermined domain and generating subsequent particles and microvoids while judging whether they are intersecting with existing ones until the predetermined particle and microvoid volume fraction is reached. Particles or voids judged to be intersecting with existing ones must be abandoned, and new ones will be generated and judged again. With the RSA method, random distribution and degradation of particles can be well fulfilled. In this study, the simulated microstructure of Al/PTFE is regenerated based on the statistical characteristics of particles and voids, as shown in Figure 2, Figure 4 and Figure 5. All processes are achieved by a MATLAB program developed by the author and the flow chart of the program is depicted in Figure 6. Reproduced simulated microstructures are depicted in Table 1 in the rightmost column, and five models were marked as 1-S, 2-S, 3-S, 4-S and 5-S, with S indicating “simulated.”

## 4. Numerical Simulation

After the real structure-based and simulated microstructures were regenerated, finite element analysis modelling a quasi-static compression test was performed with ABAQUS/Standard 6.13 (V6.11, Providence, RI, USA).

### 4.1. Material Models

To study the effectiveness of the microstructure reproduction algorithm and FE analysis methods and compare them with the experimental results, both aluminum particles and PTFE matrix are modeled as elastic–plastic materials [31]. The elastic–plastic constants are listed in Table 2.

Another important point to be considered is the properties of the materials within the microvoids. As a matter of fact, the introduction of air into composites during pressing and expansion/contraction of constituents during the sintering process is the cause of microvoids. However, in a quasi-static compression domain, the effect of air within microvoids on mechanical properties of composite is found to be small or nonexistent. The effect is due to the lack of constituents rather than the air. Thus, the microvoids are assumed to be vacuums.

### 4.2. Boundary Conditions and Homogenization Methods

For a given RVE, three types of boundary conditions are typically used: (i) the prescribed displacement boundary condition (PDBC); (ii) the prescribed traction boundary condition (PTBC); and (iii) the periodic boundary condition (PBC). The effects of the three types of boundary conditions on the prediction of RVE models were investigated by Chen C, and the results showed that PDBC and the PTBC overestimated and underestimated the yield strength, respectively, whereas the PBC provided the best performance [36]. This is because when an RVE of particle-reinforced composites is subjected to external loads, given that the size of the RVE is assumed to be very small compared with the macroscopic material, the deformation field in the RVE will be approximately the same as the field near the neighboring RVEs [37]. This statement was experimentally verified by Cheng [38]. Thus, the RVE must satisfy the conditions of (i) opposite edges deforming in an identical way and (ii) stress continuity across the boundaries. Thus, the PBC would be the best choice. In this paper, the PBC is applied to all simulated and real structure-based finite element models. For any given average deformation gradient $\bar{F}$ applied to the RVE, the PBC can be represented in the following general format [39]:

(4) $$x\left(Q_{1}\right)-x\left(Q_{2}\right)=\bar{F}\left[x\left(Q_{1}\right)-x\left(Q_{2}\right)\right],V\left(Q_{1}\right)=-V\left(Q_{2}\right)$$

where *Q*_1_ represents a general node on the face of an RVE cube, and the corresponding node *Q*_2_ is at the same location on the opposite face. *V* is the force applied to the nodes. *X* and *x* denote the positions of a material point in the original and deformed configurations, respectively. The PBC is implemented by a code in the Python language developed by the author, which extracts boundary node information and constrains the nodes based on Equation (4).

To prevent rigid body movement during compression, the lower-left node is fixed in the *x*- and *y*-directions, whereas the lower-right node is fixed in the *y*-direction. To apply a compression load to the FE models, only the top-left node is given a prescribed displacement to create a 10% compression strain due to the PBCs.

To obtain the effective elastic properties of the RVE, a homogenization approach is employed by considering the heterogeneous composite in the microscale to be a homogeneous material in the macroscale [18]. Given that the PBC is applied, macro-stress and macro-strain are derived by averaging the stress and strain tensor over the volume of the RVE:

(5) $${\bar{\sigma }}_{ij}=\frac{1}{V}\int_{V}\sigma_{ij}\left(x,y,z\right)dV;\;{\bar{\varepsilon }}_{ij}=\frac{1}{V}\int_{V}\varepsilon_{ij}c\left(x,y,z\right)dV$$

where ${\bar{\sigma }}_{ij}$ and ${\bar{\varepsilon }}_{ij}$ represent the average stress and strain in the RVE, respectively, whereas $\sigma_{ij}$ and $\varepsilon_{ij}$ represent the stress and strain of one point in the RVE, respectively. With the average stress and strain, effective elastic parameters can be calculated.

### 4.3. Meshing and Analysis

Both real microstructure-based and simulated models were meshed with three-node linear plane strain triangle elements—i.e., CEP3 elements—so the analysis of all models was under the plane strain hypothesis. To apply the periodic boundary condition, the models had to be meshed with periodic elements—i.e., the same number of elements and nodes on opposite edges. Another point to consider is that the dimensions of some particles are very small, so the element size should be small enough to mesh small particles with sufficient precision. Thus, the same number of seeds was selected along opposite edges, and the models were all meshed with an element size of approximately 1 μm. Taking the 1-R model, which is the largest, for example, it contains 183,119 nodes and 364,757 elements. Compression strain was set to 10% by applying displacement in the y direction. General contact was added for all models in case the contact of materials resulted from the contraction of microvoids. After the analysis was complete, stresses and strains were extracted by a Python code to draw stress–strain curves and calculate elastic constants.

## 5. Results and Discussion

### 5.1. Contour and Stress–Strain Distribution Analysis

**Figure 7:**
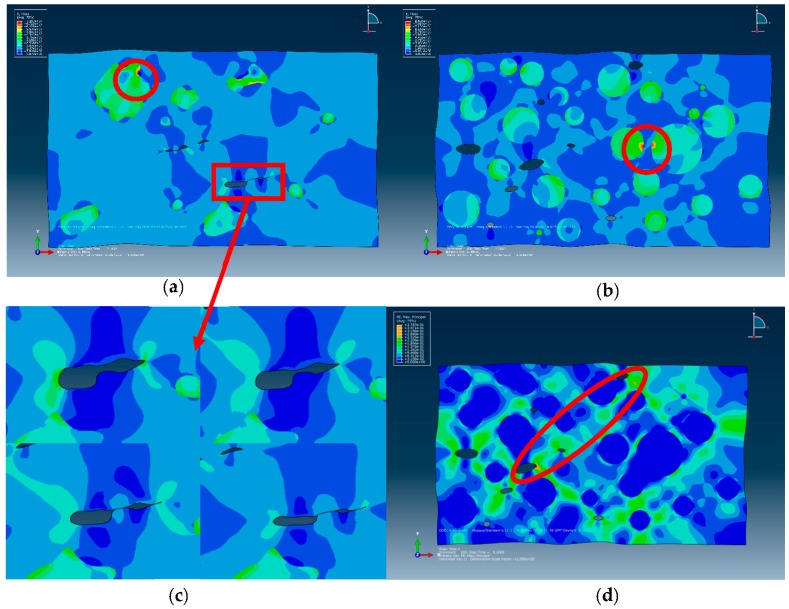
Deformation and contour of von Mises stress and plastic strain: (**a**) von Mises stress distribution of 4-R model; (**b**) von Mises stress distribution of 4-S model; (**c**) deformation of microvoid over time; (**d**) plastic strain distribution of 4-S model. Red circles indicate maximum shear stress points, and the red rectangle indicates the positions of microvoids in (**c**); red elliptical circle indicates the 45° shear bands in (**d**).

The contour plots of von Mises effective stress and deformation at the final moment are shown in Figure 7. Because of the excessive contour plots of the 10 models, 4-R and 4-S models—i.e., the models of dimensions 97 μm × 68 μm, which are also representative—are taken for example.

First, opposite edges deforming identically and stress continuity across the boundaries can be observed, which suggests that proper PBC is applied [18]. Owing to the mismatch of the elastic modulus between the particles and matrix, von Mises stress shows an inhomogeneous distribution in the matrix. When loaded, particles are subjected to higher stress than the surrounding matrix, which demonstrates the particle reinforcing mechanism. The maximum stress develops in the interfaces of the particles and matrix, especially where the particles bulge and draw close to another one (as indicated by the red circles in Figure 7a). This implies that when the composite is further loaded, bulges of particles will tend to be sheared to make more rounded ones, and the matrix will fracture owing to slippage of particles. Stress concentration occurs at tips along the direction of the long axis of microvoids owing to the close process and predicts the fracture of matrix [40]. As shown in Figure 7c, during the loading process, microvoids are closing, and shape changes are much more obvious than those of particles. This shows that the deformation mechanism within the composite is microvoids deforming and being compressed first, and then, prominent shape changes occur to the particles and matrix after the microvoids close. In addition, inhomogeneous plastic strain distribution concentrating in an array of rather straight shear bands at 45° to the loading axis can be observed in Figure 7d, indicated by the red elliptical circle; this is the main failure mechanism of Al/PTFE [35].

### 5.2. Stress–Strain Curve Comparison between Simulations and Experiments

**Figure 8:**
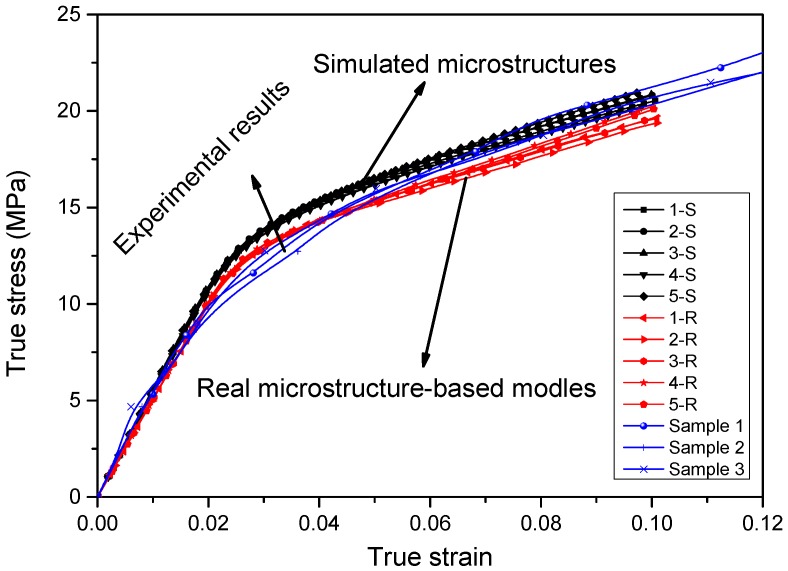
Comparison between simulated stress–strain curves and experimental results.

In this section, stress–strain curves of the five real microstructure-based and five simulated microstructure models are extracted and compared with those of the experiments, as shown in Figure 8.

Generally, as shown in Figure 8, the curves of simulated microstructure models and real microstructure-based models show good consistency. Two groups of curves show good consistency in the elastic region and within small plastic strain. When the strain is higher than 4%, obvious variation occurs. At each strain value, stresses of simulated microstructure models give higher values than real microstructure-based models. This is because there are more small particles in the simulated microstructures caused by the reproduction algorithm. When particles are being generated, there is enough space for the initial ones, which could have larger diameters. However, for the subsequent ones, they are generated after judging whether they are intersecting with existing ones; thus, only smaller particles can be generated, and even smaller ones in the following steps. Therefore, there might be less inter-particle spacing and more local particle concentration, which will enhance the strength of materials [31]. However, the consistency of each group of curves indicates that both the simulated models and real microstructure-based models are representative and, although they are selected randomly, of different dimensions.

The experimental results are shown in Figure 8. The results of both the simulated and real microstructure-based models describe the experiments satisfactorily. They are almost identical to the experimental curves in the elastic and plastic regimes. Before the strain of 0.02—although three groups of curves nearly completely overlap, the experimental curves show smaller gradients in the elastic region than the simulated models and are much closer to those of the real microstructure-based models. However, in the vicinity of the yield point, the curves of simulations deviate from the experimental ones. In the plastic region, especially when the strain is higher than 0.05, two of the experimental curves lie between those of the real microstructures and simulated microstructures, whereas one overlaps or lies above the simulated ones. The reasons for this are that, although microvoids are considered in both the simulated microstructure and real microstructure-based models, only those that are relatively large and obvious and those presented in the SEM images can be counted and used in the statistics. Moreover, the simulated microstructure models are idealized. The differences between curves can be attributed to these reasons.

**Figure 9:**
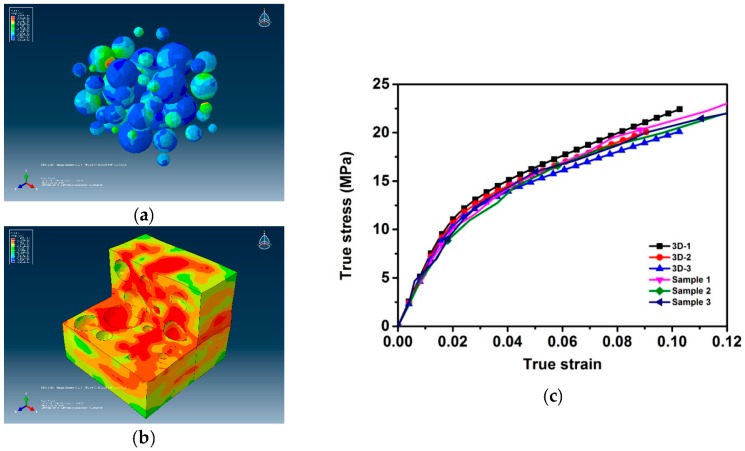
Contour plot at the final moment and stress–strain curves: (**a**) contour plot of matrix; (**b**) contour plot of particles; (**c**) comparison between stress–strain curves of 3D models and experimental results.

To compare them with the 2D models, 3D models are established based on the same statistical information. However, the largest challenge of using either a 3D real microstructure-based model or simulated model is that many elements and a highly refined mesh are required to conform to the heterogeneous nature of the microstructure. Although a 3D model without simplification of the microstructure certainly gives a better prediction of composite properties, it requires extreme computational power. Three-dimensional models were calculated previously by the author; a 50 μm × 50 μm × 50 μm model based on the statistics of particles and microvoids given in Section 3.2 cost two times more computation time than that of the 1-R and 1-S models and three times the time to extract the results. Given that the 50 μm × 50 μm × 50 μm model takes limited particles and microvoids, the author built a 100 μm × 100 μm × 100 μm model that contained approximately 500 particles and cost 18 h to compute; the output database was approximately 18 GB. The results of three 50 μm × 50 μm × 50 μm models are plotted in Figure 9c to compare with the experimental results and show the advantages of 2D models. A contour plot of the 3D-1 model at the final moment and stress–strain curves are depicted in Figure 9. In addition, identical deformation in opposite faces and stress continuity can be observed, which indicates that the periodic boundary conditions are applied. Stress concentration, shear bands and compression of microvoids can be observed and follow the same mechanism described in Section 5.1. Stress–strain curves agree well with that of the experimental ones, both in elastic and plastic regions. However, the curves show relatively large variations. This is because the 3D models are of dimensions of 50 μm × 50 μm × 50 μm, and only a limited number of particles can be considered; i.e., the 3D models are not representative. Every time particles are generated, they are of different diameters and distributions and thus have important effects on the mechanical behavior. Larger models with more particles might reduce the variation of stress–strain curves.

The elastic modulus and yield stresses of all simulations and experiments are listed in Table 3. After comparison, we can see that both the elastic modulus and yield stress calculated from the simulations are higher than those of the experiments; i.e., the simulations overestimate the elastic modulus and yield stress for the Al/PTFE studied in this paper. Here, relative errors of simulated elastic modulus and yield stresses over experimental ones were calculated to show the accuracy. Relative error s were calculated by Equation (6):

(6) $$Relative\;error=\frac{simulated\;value-experimental\;value}{experimental\;value}\times 100\%.$$

By comparison, the relative errors of predictions from 2D models over the experimental results are within 11%, whereas those of real microstructure-based models are only 1.9% and 6.6%, respectively. Corresponding predictions from 3D models show the highest variations in elastic modulus with 13.6% and 7.3% relative error over elastic modulus and yield stress, respectively. After comprehensive comparisons, real microstructure-based models that consider particle distribution, geometry and microvoids give the best results, followed by the simulated-microstructure models and 3D models based on statistics of particles and microvoids.

## 6. Conclusions

Two-dimensional microscale finite element analyses of Al/PTFE composite are conducted in this paper, which include real microstructure-based models established following a series of image processing and finite element modelling procedures and simulated microstructure models reproduced on a statistical basis considering the geometry and distribution of microvoids. In addition to 2D models, 3D finite element modelling and experiments are conducted to compare with the two types of 2D models, and the results with different methods are discussed and analyzed. Specifically,

- the real microstructure-based models, established by processing SEM images and extracting edges of Al particles and microvoids, are able to accurately represent the distribution and geometry of particles and microvoids;
- the simulated microstructure models are generated by statistics on the distribution and geometry of particles and microvoids and consider the drawbacks of RVEs regarding regular particle arrangement and micro defects in composite structures;
- compared with 3D models, the 2D real microstructure-based models and simulated microstructure models are more efficient methods to simulate the mechanical behavior of composites at the microscale;
- experimental results show that the microscale modelling of real microstructure-based models and simulated microstructure models gives good predictions of elastic modulus and yield stress. Two types of models predict the elastic modulus with relative errors of 1.9% and 10.9%, respectively, whereas those of the yield stress are 6.6% and 10.6%, respectively.

## Figures and Tables

**Table 1:** Microstructures of Al/PTFE (polytetrafluoroethylene) including real microstructures obtained by SEM, real microstructure-based models after image processing and simulated microstructures. Al particles and microvoids are represented by white hollow geometries and black solid geometries, respectively.

Order	Real Microstructures Obtained by SEM	Real Microstructures	Simulated Microstructures
1	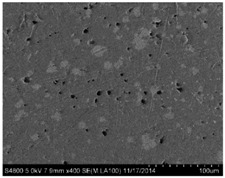	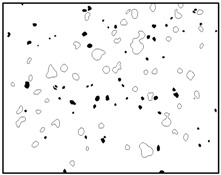	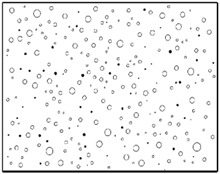
2	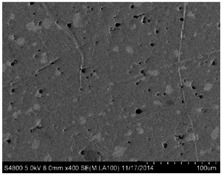	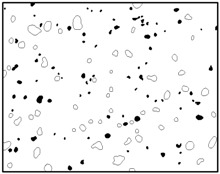	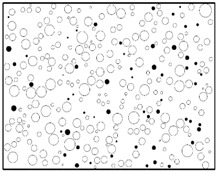
3	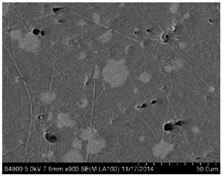	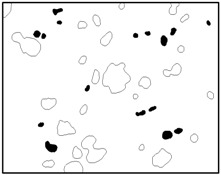	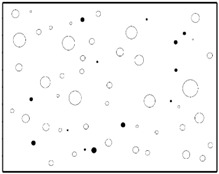
4	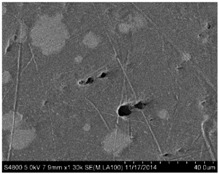	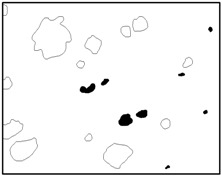	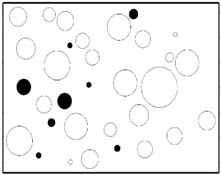
5	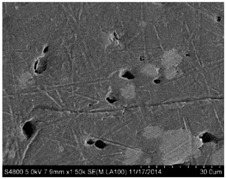	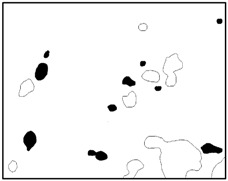	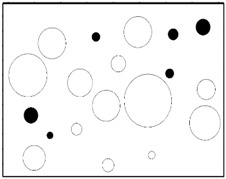

**Table 2:** Material properties of Al and PTFE used for simulation [34,35].

Material	Elastic Modulus (GPa)	Poisson’s Ratio	Yield Stress (MPa)
Al	70.00	0.33	325.00
PTFE	0.315	0.41	8.27

**Table 3:** Comparison of elastic modulus and yield stress between simulations and experiments. SE represents standard error.

Type of Models or Experiments	Dimensions of Model (μm)	Elastic Modulus (MPa)	Yield Stress (MPa)	Elastic Modulus (MPa)		Yield Stress (MPa)	
				Mean Value ± SE	Relative Error Over Experimental	Mean Value ± SE	Relative Error Over Experimental
1-S	316 × 221	544.54	13.27	548.28 ± 3.39	10.9%	13.28 ± 0.05	12.85 ± 0.27
2-S	316 × 221	551.95	13.40				
3-S	140 × 98	552.71	13.38				
4-S	97 × 68	536.80	13.11				
5-S	84 × 59	555.42	13.22				
1-R	316 × 221	502.26	12.79	504.10 ± 1.13	1.9%	10.6%	7.3%
2-R	316 × 221	505.97	12.76				
3-R	140 × 98	500.70	12.41				
4-R	97 × 68	506.62	12.93				
5-R	84 × 59	504.95	12.95				
3D-1	50 × 50 × 50	592.03	13.32	561.71 ± 15.87	13.6%	12.77 ± 0.10	11.98 ± 0.27
3D-2	50 × 50 × 50	554.71	12.84				
3D-3	50 × 50 × 50	538.40	12.39				
Sample 1	Φ 12.00 × 10.00 mm	524.36	11.96	491.51 ± 18.43	-	6.6%	-
Sample 2	Φ 12.00 × 10.00 mm	460.61	11.52				
Sample 3	Φ 12.00 × 10.00 mm	489.57	12.45				
